# Synergetic downregulation of 67 kDa laminin receptor by the green tea (*Camellia sinensis*) secondary plant compound epigallocatechin gallate: a new gateway in metastasis prevention?

**DOI:** 10.1186/1472-6882-12-258

**Published:** 2012-12-18

**Authors:** Jakob Müller, Michael W Pfaffl

**Affiliations:** 1 Physiology Weihenstephan, Technische Universität München, Research Center for Nutrition and Food Science, Weihenstephaner Berg 3, 85350 Freising, Germany

**Keywords:** Cancer, EGCG, IPEC-J2, miRNA, RNA interference

## Abstract

**Background:**

In traditional Chinese medicine, green tea is considered to have a life-prolonging effect, possibly as a result of its rich content of antioxidant tea polyphenols, and hence has the potential to prevent cancer. This study investigated the role of the major tea secondary plant compound epigallocatechin gallate (EGCG) for its inhibitory effects on the metastasis-associated 67 kDa laminin receptor (67LR).

**Methods:**

To clarify the impact of EGCG on siRNA-silenced expression of 67LR, we applied an adenoviral-based intestinal *in vitro* knockdown model, porcine IPEC-J2 cells. Quantitative real-time polymerase chain reaction was performed to analyze 67LR gene expression following treatment with physiological and pharmacological concentrations of EGCG (1.0 g/l, 0.1 g/l, 0.02 g/l and 0.002 g/l).

**Results:**

We report co-regulation of EGCG and 67LR, which is known to be an EGCG receptor. siRNA selectively and highly significantly suppressed expression of 67LR under the impact of EGCG in a synergetic manner.

**Conclusions:**

Our findings suggest that 67LR expression is regulated by EGCG via a negative feedback loop. The explicit occurrence of this effect in synergy with a small RNA pathway and a plant-derived drug reveals a new mode of action. Our findings may help to provide insights into the many unsolved health-promoting activities of other natural pharmaceuticals.

## Background

Studies assessing the beverage brewed from the leaves of the tea plant *Camellia sinensis* and its health-promoting effect are almost innumerable. It is assumed that among other constituents, the major effector of ‘green’ tea is the polyphenol epigallocatechin gallate (EGCG). One liter of green tea in a common preparation (1 g tea/100 ml water) contains approximately 300–1000 mg of this secondary plant compound [1,2]. Therewith, it provides the major share of all tea catechins. The role of green tea, and particularly EGCG, as part of a cancer-preventive diet has been well researched [3-5]. Molecular targets of the green tea polyphenols are proposed [6-8], but solid knowledge about the underlying anticancer mechanisms is still missing. In 2004, a new candidate associated with EGCG was revealed [9]: the 67 kDa laminin receptor (67LR). Tachibana et al. showed that this protein specifically binds EGCG among other tea catechins. Furthermore, a transmembrane signaling pathway was postulated in which EGCG induces the activation of myosin phosphatase through 67LR and hence develops its anticancer potential via inhibition of cell proliferation [10].

Indeed, in the scope of cancer research, 67LR has been well studied [11]. It is well known that this protein is overexpressed on the surface of cancer cells [12-15]. Thus, the involvement of 67LR in metastasis formation has been highly investigated. During this process, an invading tumor cell binds to its target tissue mediated by the local surface proteins. As laminin is a major component of the basement membrane, it was shown that enhanced expression of 67LR promotes metastasis [16]. This can be explained by the whole mechanism of the tumor invasion process. After the attachment of the circulating cancer cell, a rearrangement of the basement membrane occurs. Thereby one determining factor is the binding of laminin to 67LR. As a local consequence, laminin undergoes conformational changes and can subsequently be degraded by proteases [17]. Thereafter, the tumor cell can invade the target tissue. While examining the role of 67LR in target tissue binding during metastasis it could be shown that a 67LR-derived peptide called peptide G increases the metastatic potential in cell culture experiments [18]. Considering the fact that peptide G represents the laminin binding site of 67LR, this underlines the invasion model. In contrast, a synthetic laminin pentapeptide (YIGSR) corresponding to the binding domain of laminin was assigned as a competitive metastasis inhibitor [19].

Taken together, it becomes clear that downregulation of 67LR provides a gateway for metastasis prevention. In the work of Chen et al. this basic effect has already been demonstrated [20]. Considering the multiple clues for the cancer-preventative effect of green tea, and regarding the interaction of EGCG and 67LR, we focused on the question as to whether tea catechin can influence the expression of 67LR in the gastrointestinal tract. We conducted qRT-PCR expression analysis combined with an RNAi-knockdown model to evaluate the anti-metastatic potential of EGCG via modulation of 67LR expression *in vitro* using the porcine intestinal epithelial cell line IPEC-J2.

## Methods

### Knockdown assays

The jejunal-derived JPEC-J2 cells [21] were maintained in Dulbecco’s modified eagle medium (DMEM)/Ham’s F-12 (1:1) (PAA, Pasching, Austria) supplemented with 5% fetal bovine serum (FBS; PAA), 2 mM L-glutamine (PAA) and 100 U/ml penicillin/streptomycin (PAA) at 37°C, 100% humidity and 5% CO_2_. This cell line was kindly provided by Dr Karsten Tedin (FU Berlin). Only cells that had been passaged ten times or less were used. The cell culture assay was designed on multiwell tissue culture plates (48 W) from Greiner Bio-One (Frickenhausen, Germany) with a substrate area of 1 cm^2^. In these, 5 × 10^4^ IPEC-J2 cells were seeded in 500 μl of cell culture media, and after 48 h of settlement the desired knockdown treatment was applied together with fresh media. For gene knockdown, we used adenoviral vectors which induce siRNA expression in the target cell line in an optimized multiplicity of infection (MOI) of 400 [22]. We obtained the methodology for the gene silencing technology from SIRION-BIOTECH (Martinsried, Germany), with which we produced an individual knockdown virus targeting 67LR transcript [NCBI accession number: NM_001037146], termed ‘67lr-KD’ in this manuscript. A second targeting knockdown was designed as a positive control, designated ‘control-KD’, and targeted IKBKB [NCBI accession number: NM_001099935]. A third knockdown served as negative control and represented a non-targeting virus (named ‘NV”’). The NV was identical to the two targeting knockdown viruses (overall named ‘TV’) but was unable to induce RNA interference due to leakage of a siRNA-insert. This negative control solely evoked the effects accompanying viral infection. At 48 h post-infection, we treated the cells with different concentrations (1.0 g/l, 0.1 g/l, 0.02 g/l, 0.002 g/l) of EGCG (Sigma-Aldrich St. Louis, USA) diluted in 400 μl fresh media, following washing with 600 μl of prewarmed PBS (pH 7.5, without Ca & Mg; PAA). Here, EGCG treatment is abbreviated to ‘EGCG’, and the corresponding media control to ‘MEDIA’. For the assay layout, EGCG treatment and the media control were combined with a targeting virus or the non-targeting virus control, respectively. For the targeting viruses, both variants (targeting 67LR and the positive control) were applied. The resulting assay layout consisted of the following treatment groups: MEDIA/NV, MEDIA/TV, EGCG/NV & EGCG/TV; with TV = 67lr-KD or control-KD. All treatment variations were performed in triplicate (three cell culture wells). Cells were harvested for total RNA extraction 6 h after EGCG treatment. This included another washing step of the cell layer with PBS, followed by direct cell lysis with 350 μl of RLT buffer (RNeasy Mini Kit, Qiagen, Hilden, Germany). RNA was prepped according to the manufacturers’ instructions and then diluted to 10 ng/μl (NanoDrop ND-1000 spectrophotometer, PEQLab Biotechnologie GmbH, Erlangen, Germany) for subsequent gene expression analysis.

### Gene expression analysis

Prior to gene expression analysis by quantitative real-time polymerase chain reaction (qRT-PCR), the RNA integrity was checked using a RNA nano LABchip on an Agilent 2100 Bioanalyzer (Agilent, Santa Clara, United States). Samples were prepared using a SuperScript III Platinum SYBR Green One-Step qRT-PCR Kit (Invitrogen, Karlsruhe, Germany), and analyzed using a Corbett Rotorgene 3000 (Corbett Life Science, Sydney, Australia). The sample volume of 10 μl contained 38 ng RNA, 5 μl SYBR-Mix and 10 pmol primer mix (67LR [NCBI accession number: NM_001037146], forward primer: AGCGAGCTGTGCTGAAGTTT & reverse primer: GTGAGCTCCCTTGTTGTTGC; IKBKB [NCBI accession number: NM_001099935], forward primer: GGCGAACAGAGATTAATACACAAA & reverse primer: GTGCCGAAGCTCCAGTAGTC). The cycler program included a 10 min reverse transcription reaction at 50°C followed by a 5 min denaturation step at 95°C. Subsequently, under continuous fluorescence measurement, 40 amplification cycles (denaturation 95°C/10 s, annealing 60°C/10 s, extension 72°C/15 s) were performed, followed by a terminal melting curve measurement from 40°C to 95°C (in 0.5°C steps with a lag time of 2 sec). All qRT-PCR experiments were performed in duplicate (two one-step qRT-PCRs). The raw data were obtained using Rotor-Gene 6 software (Corbett Life Science, Sydney, Australia) via the implemented comparative quantification algorithm. Furthermore, the melting curve analysis of the Rotor-Gene 6 software was consulted for control of primer specificity. Relative changes in gene expression (shown as percentages) were determined under the terms of the ^ΔΔ^Cq method [23]. The Cq value displayed for the cycle number was required by the fluorescence signal of a single qPCR sample to cross a predefined threshold [24,25]. The chosen reference genes were Histone H3 ([NCBI accession number: XM_003356519], forward primer: ACTGGCTACAAAAGCCGCTC, and reverse primer: ACTTGCCTCCTGCAAAGCAC) and GAPDH ([NCBI accession number: XM_003358301], forward primer: AGATCCAGGATAAGGAAGGCA, and reverse primer: GCTCCACCTCCAGGGTGAT). All primers were generated by Eurofins MWG Operon (Ebersberg, Germany).

### Statistical data evaluation

The plots visualizing gene expression indicate mean values from repeated measurements. Thereby, the replicates are specified as “n = (a) × (b) × (c)”. These variables stand for the number of assays included (a), the cell culture replicates (b) and the qRT-PCR reactions conducted (c). Significant changes through treatments were ascertained with paired *t*-tests (SPSS 19.0, IBM Corporation, Armonk, USA), and resulting *P*-values are indicated by asterisks, as follows: *P* ≤ 0.001 = extremely significant (***), *P* ≤ 0.01 = highly significant (**), *P* ≤ 0.05. = significant (*), *P* > 0.05 = not significant (n.s.). Variations between different EGCG concentrations where tested for significance by applying a, one-way ANOVA (SPSS 19.0, IBM Corporation, Armonk, USA). The letters a, b, and c indicate extremely significant intermediate variances (*P* ≤ 0.001). The assay data was additionally analyzed by principal component analysis (PCA) using GenEx software (version 5.3.2.13, MultiD, Göteborg, Sweden). Therefore, mean centered ^Δ^Cq values were applied [26]. PCA is a statistical visualization method for multivariable data sets, which reduces the dimensionality while exposing the maximal variation embedded [27].

## Results and discussion

**Figure 1:**
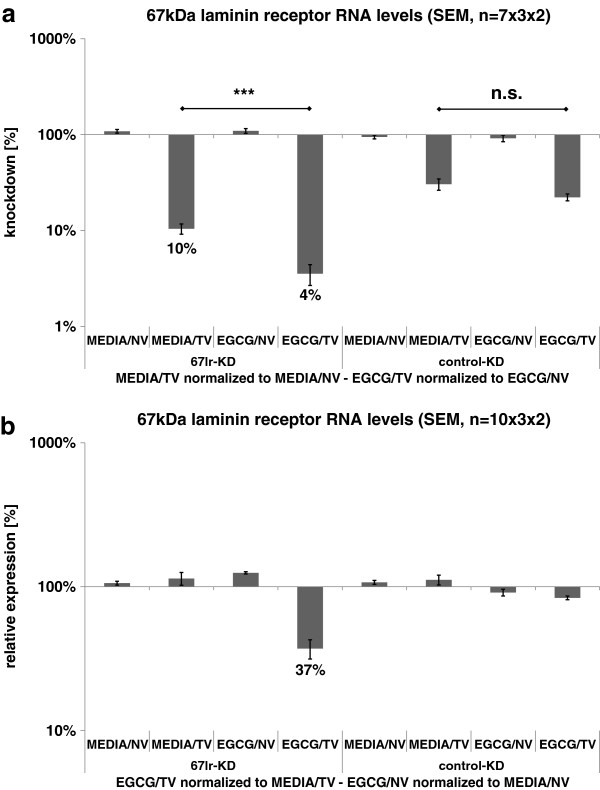
**Knockdown assays comparing 67lr-KD and control-KD under EGCG treatment. a**) Pooled data from seven assays normalized to the non-targeting virus samples. This normalization revealed net knockdown effects. **b**) Pooled data from ten assays normalized to the media control samples. This normalization revealed the net regulation induced by the drug treatment. (Legend: EGCG = EGCG treatment, MEDIA = media control, NV = non-targeting virus, TV = targeting virus, 67lr-KD = 67LR knockdown, control-KD = control knockdown).

In our cell culture assays, we combined pairwise drug treatments (EGCG *vs*. media control) with knockdowns (target genes *vs*. control virus). Thereby, the relative gene regulation (calculated by 2^-ΔΔCq^ given as percentage relative to control) caused by a combinatory treatment with target virus and/or EGCG was evaluated for every single treatment variant in reference to its particular control group. By this method, the net knockdown efficiency was calculated by normalizing target knockdown-treated samples containing one individual concentration of EGCG to the samples containing the same EGCG concentration but that were treated with the non-target control virus. In the same manner, the net effect of a drug treatment becomes clear when it is normalized within one virus treatment group to its media control partner. Figure 1 shows the mean values from repetitions of our cell culture assay, thereby data in Figure 1a was offset against the knockdown controls within one treatment (EGCG or media) variant to obtain the net knockdown on the corresponding target gene. The plot shows that the resulting downregulation was approximately 90% in wells treated solely with medium and the target virus for 67LR (MEDIA/TV, 67lr-KD) (mean of seven independent experiments). Interestingly, 67lr-KD efficiency was altered when EGCG was applied instead of pure media (EGCG/TV, 67lr-KD). The gene-silenced cells that were co-treated with EGCG showed an additional reduction in 67LR expression to result in a knockdown efficiency of 96%. At first, the step from 90% to 96% seems less impressive, but on closer analysis it represents a large change. For all expression plots, the value is presented on a logarithmic scale. Regarding the fact that relative change in mRNA expression is calculated, a remaining transcript level of 10% must be reduced by more than 50% to fall down to 4%.

In follow-up measurements, we clarified if this observation originated from an artifact resulted from the methodology applied. Therefore, we performed the assay with another target-knockdown, which did not target 67LR (shown in Figure 1a as ‘control KD’). In this setting, EGCG had no significant effect on enhancing the knockdown efficiency, leading to the assumption that the combination of 67LR protein and its binding partner EGCG can promote synergetic downregulation. To demonstrate the co-action between 67LR and EGCG, the data underlying Figure 1a was adjusted to the media control within one knockdown scenario in the plot from Figure 1b. Thus, the extent of the synergetic effect evoking 63% of relative residual 67LR mRNA expression as a mean value from ten independent experiments was revealed. In contrast the other treatment combinations where EGCG was applied in absence of the 67LR knockdown did not lead to substantial changes in relative expression.

**Figure 2:**
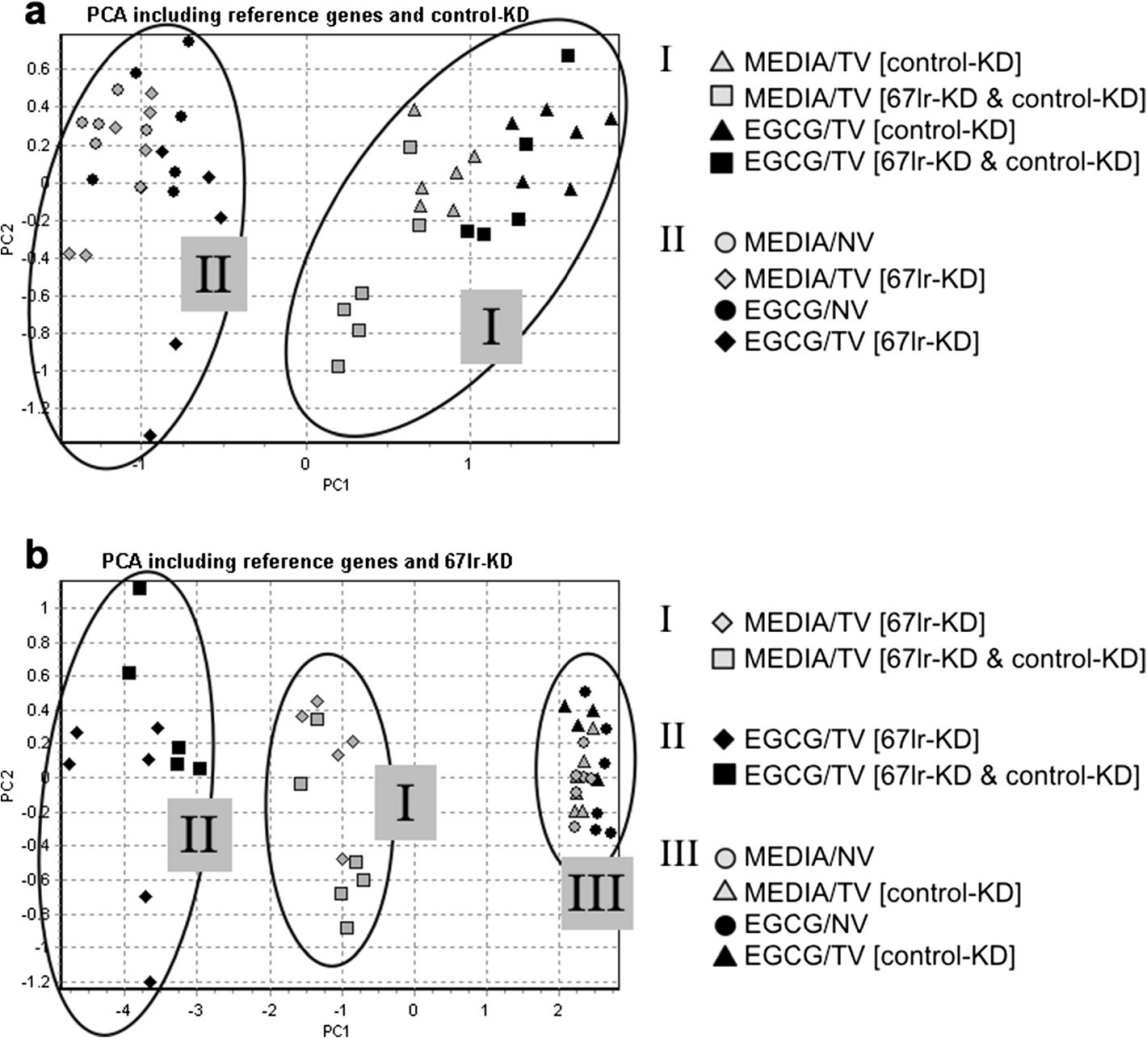
**Principal component analysis comparing the influence of EGCG on 67LR versus the control gene. a**) For the control gene, the impact of the drug treatment was dependent on whether a gene knockdown was applied (I) or not (II). **b**) For the 67LR gene, siRNA-induced downregulation altered the extent of its synergy with EGCG (I) and a new data cluster was separated from the samples solely treated with the 67lr-KD (II). When no knockdown was applied, EGCG did not affect the gene regulation (III) in accordance with Figure 3a. (Color code: black labels indicate samples containing EGCG, grey labels indicate no EGCG; Legend: EGCG = EGCG treatment, MEDIA = media control, NV = non-targeting virus, TV = targeting virus, 67lr-KD = 67LR knockdown, control-KD = control knockdown).

To confirm our observations independent of normalization to any control groups (virus/drug), we consulted principal component analysis (PCA). With this statistical data evaluation it is possible to analyze the outcome of an assay by sample [26]. The PCA considers the collective variation in gene expression an RNA sample bears in reference to all genes measured from it. Therefore, our expression data were inserted in the PCA as raw data solely corrected against the reference genes but not against any control groups of the underlying cell culture assay. The findings from this independent kind of data analysis were in accordance with those from the relative fold changes shown in Figure 1a and b. The PCA-plot in Figure 2 shows data from an assay that also includes eight treatment variants (MEDIA/NV, MEDIA/TV, EGCG/NV & EGCG/TV; with TV = 67lr-KD and/or control-KD) each replicated six times (3 cell culture wells × 2 qRT-PCR reactions). For each of these 48 datapoints (samples), the gene expression of two reference genes and two target genes (67lr-KD, control-KD) was ascertained. In Figure 2a, the gene expression data including the reference genes and the control-KD gene was analyzed. The resulting image of the PCA shows data clustering in two clouds: (I) with (MEDIA/TV [control-KD] & EGCG/TV [control-KD]) or (II) without (MEDIA/NV, EGCG/NV & MEDIA/TV [67lr-KD], EGCG/TV [67lr-KD]) the considered control KD. In contrast, the PCA considering the reference genes and the 67LR knockdown (67lr-KD) (Figure 2b) reveals three data clusters: (I) the 67kd-KD combined with medium treatment (MEDIA/TV [67lr-KD]), (II) the 67kd-KD combined with EGCG treatment (EGCG/TV [67lr-KD]) and (III) samples not containing the considered 67LR-knockdown but treated with either media or EGCG (MEDIA/NV, EGCG/NV & MEDIA/TV [control-KD]). Thus, the additional separation into a third cloud is again induced exclusively under the synergy of 67lr-KD and EGCG.

**Figure 3:**
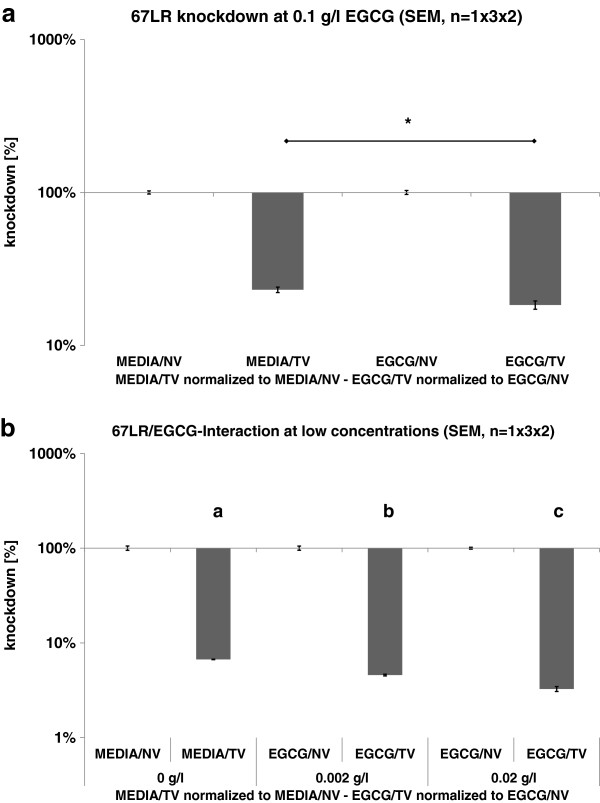
**67LR/EGCG-synergy when the secondary plant metabolite was added at potencies below the actual EGCG-content of freshly brewed green tea.** (Legend: EGCG = EGCG treatment, MEDIA = media control, NV = non-targeting virus, TV = targeting virus, 67lr-KD = 67LR knockdown, control-KD = control knockdown).

The next question was to ascertain if EGCG was able evoke the same effect at concentrations lower than that measured in freshly brewed green tea [1,2]. Therefore, we added EGCG at concentrations from one (0.1 g/l) up to three potencies (0.02 g/l & 0.002 g/l) below the actual tea content (Figure 3). Again, we observed 67LR/EGCG synergy, but this effect was less distinct. At an EGCG concentration of 0.1 g/l, we observed a 3.7-fold reduction in downregulation of 67LR (Figure 3a), and at 0.02 g/l & 0.002 g/l, we were still able to measure a significant effect (*P* = 0.001; Figure 3b).

Since the discovery of intra- and intercellular short ribonucleic acids, many different species of non-coding but functional active small RNAs have been identified [28]. Among these, miRNAs and siRNAs are involved in negative gene regulation. However, with increasing knowledge from *in vitro* studies, pathways that once initially seemed distinct have become blurred, revealing the extent of the complexity as to how small RNAs act in gene regulation. miRNAs were originally described to bind the 3^′^UTR alone, but there is now evidence that miRNAs can also act upon the coding region of a transcript [29]. Within the complex machinery of posttranscriptional gene regulation, it has been shown that certain miRNA signatures can serve as biomarkers for oncology [30]. Thus, small RNA synthesis or leakage can affect cancer and influence metastasis positively or negatively depending on the targeted gene.

Our results indicate EGCG significantly influences siRNA-induced downregulation of 67LR expression. Since a close relation of this protein and its transcript has been ascertained [31], these findings show a correlation between the plant compound and its receptor [9] via a negative feedback loop. Reasonably the anti metastatic potential of green tea as a daily diet develops best outgoing from the gastrointestinal tract. In regard to its carcinogenic potential, enhanced expression levels of 67LR have been associated with metastasis formation in different tissues, including the intestine [32].

Considering our findings that EGCG acts synergistically and promotes siRNA-induced downregulation of 67LR protein, a new concept of how secondary plant metabolites act in cancer prevention can be established. The complex feedback control system of miRNA regulation of oncogenes might be influenced selectively in synergy with plant compounds, and may provide clues to the potential anti-carcinogenic mechanism of EGCG [2].

## Conclusions

Metastasis presents the largest challenge in terms of mortality in the treatment of cancer. In this study, we identified a significant correlation of EGCG co-regulation with siRNA-silenced expression of 67LR in a porcine intestinal cell line. As pigs are monogastric animals, IPEC-J2 cells represent a model conferrable to the human gastrointestinal tract. As part of a cancer-preventive diet, green tea may act via synergic action of small RNA regulation and the properties of an herbal-derived drug, EGCG.

Future tasks are represented by the assessment of the 67LR within the small RNA regulatory network of metastasis models and hence the clarification of its role as an endogenous miRNA target. Evaluating the reproducibility of the shown synergic effect in other *in vitro* models may help to provide clues as to the mode of action of pharmacologically-applied plant compounds.

## Abbreviations

3^′^UTR: Three prime untranslated region; 67LR: 67 kDa laminin receptor; EGCG: Epigallocatechin gallate; MOI: Multiplicity of infection; PCA: Principal component analysis; RNAi: RNA interference; RT-qPCR: Quantitative reverse transcription real-time polymerase chain reaction; siRNA: Small interfering RNA; EGCG: EGCG treatment; MEDIA: Media control; NV: Non-targeting virus; TV: Targeting virus; 67lr-KD: 67LR knockdown; control-KD: Control knockdown.

## Competing interests

The authors declare that they have no competing interests.

## Authors’ contributions

The assays in this study where designed and conducted by JM. The data evaluation was performed by JM. The manuscript was drafted by JM and supervised by MP. Both authors read and approved the final manuscript.

## Pre-publication history

The pre-publication history for this paper can be accessed here:

http://www.biomedcentral.com/1472-6882/12/258/prepub
